# Supplementary material for: Attitudes, beliefs, and practices regarding complementary and alternative medicine use: Influenza vaccine intake
Source: PLoS One. 2025 Jul 23;20(7):e0320412. doi: 10.1371/journal.pone.0320412 (PMC12286328; doi:10.1371/journal.pone.0320412)
Supplement: S1 Table — (DOCX) [file pone.0320412.s001.docx]

S1 Table: Beliefs and Practices About Herbal and Alternative Therapies for Prevention and Treatment of Respiratory Infections.

|  | Strongly disagree | Disagree | Neutral | Agree | Strongly agree | Positive practice |
| --- | --- | --- | --- | --- | --- | --- |
| 1. Intake of vitamin C helps increase immunity and reduce the chances of developing Respiratory infection |  | 11(3.0%) | 30(8.0%) | 169(46.7) | 153(42.3) | 322(89.0%) |
| 1. Vitamin D helps in improving immunity, which may reduce the risk of the common cold | 1(0.3%) | 29(8.0%) | 84(22.9%) | 163(45.0%) | 86(23.8%) | 249(68.8%) |
| 1. Do you think taking a ginger and honey mixture helps in preventing the chances of developing flu and cough | 2(0.6%) | 19(5.2%) | 68(18.5%) | 176(48.6%) | 98(27.1%) | 279(75.7%) |
| 1. Gargling with a solution of warm salt water is the best way to battle sore throat by killing germs and viruses | 6(1.7%) | 38(10.5%) | 99(27.1%) | 140(38.7%) | 80(22.1%) | 220(60.8%) |
| 1. Supplementing 1 spoon full of apple cider vinegar (ACV) mixed with warm water helps increase immunity and reduce the chances of developing respiratory infection | 4(1.1%) | 46(12.7%) | 165(45.3%) | 104(28.7%) | 44(12.2%) | 148(40.9%) |
| 1. Drinking plenty of clean water helps in controlling the dehydration associated with symptoms of respiratory (flu, cough, sore throat) | 3(0.8%) | 14(3.9%) | 52(14.1%) | 162(44.8%) | 132(36.5%) | 294(81.2%) |
| 1. The use of turmeric in a daily routine will help strengthen immunity against respiratory infection | 3(0.8%) | 26(7.2%) | 163(44.8%) | 121(33.4%) | 50(13.8%) | 171(47.2%) |
| 1. Do you think that eating garlic helps to increase immunity and reduce the chance of developing a respiratory infection? | 1(0.3) | 19(5.2%) | 73(19.9%) | 165(45.6%) | 105(29.0%) | 270(74.6%) |
| 1. Do you think that eating onions (or onion peel) help to increase immunity and reduce the chance of developing a respiratory | 4(1.1%) | 26(7.2%) | 117(32.0%) | 151(41.7%) | 65(18.0%) | 216(59.7%) |
| 1. Do you think that eating fish oil known as omega-3 helps to increase immunity and reduce the chance of developing a respiratory infection? |  | 34(9.4%) | 107(29.3%) | 164(45.3%) | 58(16.0%) | 222(61.3%) |
| 1. Do you think that vitamins and herbal supplements treat/ reduce the incidence of respiratory infection? | 2(0.6%) | 16(4.4%) | 80(21.8%) | 190(52.5%) | 75(20.7%) | 265(73.2%) |
| 1. Do you think that consuming honey and lemon tea helps increase immunity and reduce the chance of developing flu or cough and sore throat? | 2(0.6%) | 14(3.9%) | 55(14.9%) | 184(50.8%) | 108(29.8%) | 292(80.7%) |
| 1. Do you think that consuming costus roots helps increase immunity and reduce the chance of developing a respiratory infection? | 5(1.4%) | 30(8.3%) | 196(53.9%) | 86(23.8%) | 46(12.7%) | 132(36.5%) |
| 1. Do you think that eating black seeds (Prophetic medicines) helps increase immunity and reduce the chance of developing a respiratory infection? |  | 16(4.4%) | 135(37.0%) | 136(37.6%) | 76(21.0%) | 212(58.6%) |
| 1. Steam inhalation (using essential oils) is the greatest way of preventing/or killing the respiratory infection | 9(2.5%) | 26(7.2%) | 100(27.3%) | 156(43.1%) | 72(19.9%) | 228(63.0%) |
