# Supplementary material for: Attitudes, beliefs, and practices regarding complementary and alternative medicine use: Influenza vaccine intake
Source: PLoS One. 2025 Jul 23;20(7):e0320412. doi: 10.1371/journal.pone.0320412 (PMC12286328; doi:10.1371/journal.pone.0320412)
Supplement: S1 File — (DOCX) [file pone.0320412.s002.docx]

**Attitudes, Beliefs, and Practices Regarding Complementary and Alternative Medicine use: Influenza Vaccine intake**

# Authors

Dala N. Daraghmeh^1^, Ahmad Salah^1^, Nasim Hamdan^1^, Abdallah Zamareh^1^

# Affiliation

1. Faculty of Pharmacy, Al-Quds University, P.O. Box 20002, Jerusalem, Palestine.

# Corresponding author

Address correspondence to Dala Daraghmeh. Email: [dadaraghmeh@gmail.com](mailto:dadaraghmeh@gmail.com), [**https://orcid.org/0000-0003-1933-5241**](https://orcid.org/0000-0003-1933-5241)**.**

# **Supplementary File1: Survey on CAM Use and Beliefs in Influenza Management**

# Section 1: Socio-demographic data

| 1. Age? | _______ |
| --- | --- |
| 1. Gender? |  |
| Male | ❑ |
| Female | ❑ |
| 1. What is the highest level of education you completed? |  |
| Less than bachelor degree | ❑ |
| Bachelor’s degree | ❑ |
| Master’s or Doctorate degree | ❑ |
| 1. Which income group does your household fall under (monthly income)? |  |
| Less than JD 400 | ❑ |
| JD 400 – JD1000 | ❑ |
| >JD1000 | ❑ |
| 1. What best characterizes the area where you live? |  |
| Palestinian refugee camp | ❑ |
| Village | ❑ |
| City | ❑ |
| 1. What is your current marital status? |  |
| Married | ❑ |
| Divorced | ❑ |
| Widowed | ❑ |
| Single | ❑ |
| 1. Occupation |  |
| Medical field, please specify_______ | ❑ |
| Not Medical field | ❑ |
| Unemployed | ❑ |

# Section 2: Clinical characteristics

| 1. Do you smoke? |  | |
| --- | --- | --- |
| Active smoker | ❑ |  |
| Ex-smoker | ❑ |  |
| Never smoke | ❑ |  |
| 1. How good is your health generally? |  | |
| Very good | ❑ |  |
| Good | ❑ |  |
| Bad | ❑ |  |
| Very bad | ❑ |  |
| 1. Do you have any chronic illnesses? |  | |
| Yes | ❑ |  |
| No | ❑ |  |
| 1. Do you exercise? |  | |
| No | ❑ | |
| Yes, more than once a week | ❑ | |
| 1. How many times did you get the flu this year? |  | |
| I did not get flu | ❑ | |
| Once | ❑ | |
| Twice | ❑ | |
| More than 2 | ❑ | |
| 1. What do you usually use to manage flu symptoms |  | |
| Pharmaceutical drug | ❑ | |
| Complementary and alternative medicine | ❑ | |
| Both | ❑ | |
| Neither | ❑ | |
| 1. Have you been hospitalized from influenza symptoms |  | |
| Yes | ❑ | |
| No | ❑ | |
| 1. Did you get the flu vaccine (flu shot) this year? |  | |
| Yes | ❑ | |
| No | ❑ | |

# Section 3: Complementary and alternative medicine use

| 1. **Any CAM used for Flu management** |  |
| --- | --- |
| Yes | ❑ |
| No | ❑ |
| 1. **Herbal** |  |
| Yes | ❑ |
| No | ❑ |
| 1. **Biologically-based therapies** |  |
| Yes | ❑ |
| No | ❑ |
| 1. **Manipulative and body-based methods** |  |
| Yes | ❑ |
| No | ❑ |
| 1. **Alternative medical systems** |  |
| Yes | ❑ |
| No | ❑ |
| 1. **Mind-body medicine** |  |
| Yes | ❑ |
| No | ❑ |

# Section 4: Experience and perceptions on complementary and alternative medicine use

| 1. **Are you aware of alternative or complementary medicine practices?** | |
| --- | --- |
| Yes | ❑ |
| No | ❑ |
| 1. **During the last 12 months, have you used any form of alternative or complementary medicine?** | |
| Yes | ❑ |
| No | ❑ |
| 1. **Do you intend to recommend CAM modalities to others?** | |
| Yes | ❑ |
| No | ❑ |
| 1. **How would you rate your knowledge about different alternative medicine modalities?** | |
| Very Limited Knowledge | ❑ |
| Limited Knowledge | ❑ |
| Moderate Knowledge | ❑ |
| Good Knowledge | ❑ |
| Extensive Knowledge | ❑ |

# Section 5: Belief and practice of CAM for influenza treatment and prevention

| **Do you believe in the holistic approach of alternative medicine?** | |
| --- | --- |
| Yes | ❑ |
| No | ❑ |
| **How easily can you access alternative medicine practices in your region?** | |
| Easy | ❑ |
| Moderate | ❑ |
| Hard | ❑ |
| **Have the costs associated with alternative medicine ever influenced your decision to use or continue using these practices?** | |
| Yes | ❑ |
| No | ❑ |
| **Do you believe that some of the benefits you've experienced from alternative medicine might be attributed to a placebo effect?** | |
| Yes | ❑ |
| No | ❑ |
| **your educational background influence your trust in alternative medicine practices?** | |
| Yes | ❑ |
| No | ❑ |

| **In your experience, do the benefits of alternative medicine tend to be more short-term or long-term?** | |
| --- | --- |
| A) I believe the benefits of alternative medicine are mostly short-term. | ❑ |
| B) I see both short-term and long-term benefits with alternative medicine, with a balanced effect. | ❑ |
| C) The duration of benefits in alternative medicine depends on the specific health condition being addressed. | ❑ |
| D) I believe the benefits of alternative medicine are mostly long-term. | ❑ |
| E) I don't have a strong opinion or clear observation regarding the duration of benefits in alternative medicine. | ❑ |
| **What is your perception of the effectiveness of alternative or complementary medicine compared to conventional mediine?** | |
| I believe alternative medicine is more effective than conventional medicine. | ❑ |
| I believe conventional medicine is more effective than alternative medicine. | ❑ |
| I believe both alternative and conventional medicine are equally effective. | ❑ |
| My perception varies depending on the specific health condition or situation. | ❑ |
| I have no opinion on the effectiveness of alternative or conventional medicine. | ❑ |
| **How would you describe the cost of alternative medicine compared to conventional medical treatments?** | |
| Alternative medicine is significantly more affordable than conventional treatments | ❑ |
| Alternative medicine is slightly more affordable than conventional treatments | ❑ |
| Costs are similar for alternative and conventional treatments. | ❑ |
| Conventional treatments are slightly more affordable than alternative medicine. | ❑ |
| Conventional treatments are significantly more affordable than alternative medicine. | ❑ |

# Section 6: Belief and practice of CAM for influenza treatment and prevention

|  | Strongly agree | Agree | I don’t know | Disagree | Strongly disagree |
| --- | --- | --- | --- | --- | --- |
| 1. Intake of vitamin C helps increase immunity and reduce the chances of developing Respiratory infection |  |  |  |  |  |
| 1. Vitamin D helps in improving immunity, which may reduce the risk of the common cold |  |  |  |  |  |
| 1. Do you think taking a ginger and honey mixture helps in preventing the chances of developing flu and cough |  |  |  |  |  |
| 1. Gargling with a solution of warm salt water is the best way to battle sore throat by killing germs and viruses |  |  |  |  |  |
| 1. Supplementing 1 spoon full of apple cider vinegar (ACV) mixed with warm water helps increase immunity and reduce the chances of developing respiratory infection |  |  |  |  |  |
| 1. Drinking plenty of clean water helps in controlling the dehydration associated with symptoms of respiratory (flu, cough, sore throat) |  |  |  |  |  |
| 1. The use of turmeric in a daily routine will help strengthen immunity against respiratory infection |  |  |  |  |  |
| 1. Do you think that eating garlic helps to increase immunity and reduce the chance of developing a respiratory infection? |  |  |  |  |  |
| 1. Do you think that eating onions (or onion peel) help to increase immunity and reduce the chance of developing a respiratory infection? |  |  |  |  |  |
| 1. Do you think that eating fish oil known as omega-3 helps to increase immunity and reduce the chance of developing a respiratory infection? |  |  |  |  |  |
| 1. Do you think that vitamins and herbal supplements treat/ reduce the incidence of respiratory infection? |  |  |  |  |  |
| 1. Do you think that consuming honey and lemon tea helps increase immunity and reduce the chance of developing flu or cough and sore throat? |  |  |  |  |  |
| 1. Do you think that consuming costus roots helps increase immunity and reduce the chance of developing a respiratory infection? |  |  |  |  |  |
| 1. Do you think that eating black seeds (Prophetic medicines) helps increase immunity and reduce the chance of developing a respiratory infection? |  |  |  |  |  |
| 1. Steam inhalation (using essential oils) is the greatest way of preventing/or killing the respiratory infection |  |  |  |  |  |
